# Accelerometer-measured physical activity and sedentary time in a cohort of US adults followed for up to 13 years: the influence of removing early follow-up on associations with mortality

**DOI:** 10.1186/s12966-020-00945-4

**Published:** 2020-03-14

**Authors:** Jakob Tarp, Bjørge Herman Hansen, Morten Wang Fagerland, Jostein Steene-Johannessen, Sigmund Alfred Anderssen, Ulf Ekelund

**Affiliations:** 1 Department of Sports Medicine, Norwegian School of Sports Sciences, Sognsveien 220, 0806 Oslo, Norway; 2 Department of Public Health, Sports and Nutrition, University of Agder, Kristiansand, Norway; 3 Department of Chronic Diseases and Ageing, Norwegian Institute of Public Health, Oslo, Norway

**Keywords:** Cohort study, Epidemiology, Exercise, Leisure activity

## Abstract

**Background:**

Observational studies linking physical activity with mortality are susceptible to reverse causation bias from undiagnosed and prevalent diseases. Researchers often attempt to deal with reverse causation bias by excluding deaths occurring within the first 1 or 2 years from the analysis, but it is unclear if excluding deaths within this time-frame is sufficient to remove bias.

**Methods:**

We examined associations between total and intensity-specific physical activity and sedentary time with all-cause mortality in a prospective cohort of 3542 individuals from the 2003–2006 NHANES cycles. In order to yield measures of association hypothesized as minimally influenced by reverse causation bias the primary analysis excluded individuals with < 5 years of follow-up. Accelerometer-measured physical activity was linked with recently updated vital status from the National Death Index with a median follow-up of 10.8 years.

**Results:**

Hazard ratios (95% confidence intervals) were 0.74 (0.53, 1.04), 0.52 (0.37, 0.73), and 0.61 (0.38, 1.01) for ascending quartiles of total physical activity against the least active reference. Hazard ratios for ascending moderate-to-vigorous physical activity quartiles against the reference were 0.67 (0.47, 1.96), 0.67 (0.47, 0.95), and 0.68 (0.39, 1.18). Associations for light intensity physical activity and sedentary time were smaller in magnitude and all confidence intervals included unity. Total activity and moderate-to-vigorous physical activity hazard ratios from analyses only excluding deaths within the first 2 years were inflated by 13 and 26% relative to analysis restricted to ≥5 years of follow-up.

**Conclusions:**

The pattern of associations suggested total physical activity and moderate-to-vigorous physical activity were associated with lower mortality after more than 10 years of follow-up and excluding the first 5 years of observation time to minimize the impact of reverse causation bias. Excluding deaths within the first 2 years appeared insufficient to minimize the impact of reserve causation bias.

## Introduction

Physical activity improves quality of life and is positively associated with longevity [1]. However, the majority of data informing physical activity guidelines is based on self-reported activity [1]. Device-based measurements of physical activity provide more accurate assessment of various intensities of physical activity, especially light intensity, and sporadic bouts of activity compared with self-report. Further, risk of bias from differential misclassification is greatly reduced [2, 3]. Devices-based measurements are now being increasingly implemented in prospective cohort studies. Results from these studies have confirmed the reduced risk of mortality with higher levels of at least moderate intensity physical activity and some studies have suggested that light intensity physical activity (LPA) may be sufficient to reduce mortality risk [4–7]. High levels of sedentary time have also been associated with higher mortality [7]. However, previous studies are limited by either short follow-up time or relatively low sample size and few deaths. Subclinical disease may impact physical activity patterns due to e.g. fatigue, loss of appetite or through reduced cardiopulmonary or musculoskeletal function and may result in low levels of physical activity [8], a source of error know as reverse causation bias when this condition also increases the risk of mortality. Similarly, individuals living with a chronic disease may have low physical activity levels due to their disease and this may not be fully captured by including indicator variables for health status in the statistical model [9]. One attempt to control for reserve causation bias is to restrict analyses to individuals with ≥1 or ≥ 2 years of follow-up. However, such short time may not fully remove the confounding effect of this bias [10, 11] as this approach is based on the assumption that individuals with low physical activity levels due to undiagnosed diseases will have died within just 2 years which may not be sufficiently conservative. Thereby, the magnitude of the protective association between device-measured physical activity and mortality may be inflated as low physical activity levels because of subclinical disease have not been sufficiently accounted for.

Recently, the National Center for Health Statistics updated mortality status in the National Health and Nutrition Examination Survey (NHANES) with follow-up expanding from December 2011 to December 2015 allowing analyses that substantially reduce the potential influence of reverse causation bias by eliminating a larger window of early follow-up. We therefore examined how increasing early follow-up exclusion from 0 to 5 years after baseline would influence the magnitude and shape of associations between total and intensity-specific physical activity and sedentary time with risk of all-cause mortality in the NHANES 2003–2006 cohorts.

## Methods

This study is based on data from the NHANES 2003–2006 cycles. NHANES samples noninstitutionalized U.S. civilians using a multistage probability sampling design that considers geographical area and minority representation [12]. Physical activity was measured by accelerometry (AM-7164; ActiGraph) for 7 days using a 1-min epoch. Individual level data from at least ≥10 h for ≥4 days between 6 AM and midnight was required for inclusion in analysis. We defined non-wear according to the Choi algorithm [13]. Total physical activity was defined as total recorded counts/wear-time (CPM). LPA, moderate-to-vigorous physical activity (MVPA), and sedentary time were defined as time with the following 1-min count-values; 101–1951, ≥1952 and ≤ 100, respectively [14]. Mortality-status was determined by linkage with the National Death Index through December 31, 2015 [15]. Body mass index (BMI) was calculated from measured height and weight. Other covariables were included from a standardized questionnaire. We excluded individuals < 40 years old at the examination date. From 6361 individuals ≥40 years of age who were eligible for mortality follow-up, 3860 had complete data on physical activity, covariables and mortality status (details of exclusions provided in the Additional File Figure 1).

### Statistical analysis

We used cox proportional hazards regression models to estimate hazard ratios (HR) and 95% confidence intervals (CI) for the associations between activity exposures (in quartiles, using least active as reference) sedentary time (using least sedentary as reference) and all-cause mortality with time since examination date as the time-scale. We created quartiles based on wear-time normalized activity/sedentary time levels (using % wear-time). We included the following co-variables; age (continuous), sex, wear-time (not total physical activity), BMI (continuous), education (<high School, high School (including General Education Development), or > high School), race/ethnicity (Mexican-American, Non-Hispanic White, Non-Hispanic Black, or other), alcohol consumption status (never, former, current or missing), smoking-status (never, former or current), marital status (married/living with partner or widowed/divorced/separated/never married), mobility limitations (any difficulty walking up ten steps or walking a quarter mile), number of medical conditions (continuous sum of the following conditions; diabetes, congestive heart failure, coronary heart disease, angina/angina pectoris, heart attack, stroke, cancer or malignancy). MVPA and sedentary time models were mutually adjusted (continuous form, wear-time normalized). We noticed no violation of the proportional hazards assumption in visual inspection of log-log plots and Schoenfeld residuals plotted against follow-up time. Our primary analysis was restricted to individuals with ≥5 years of follow-up. In secondary analysis, we further excluded individuals reporting mobility limitations and those with any of the medical conditions listed above. Trends across activity and sedentary time quartiles were examined by defining quartiles by their median physical activity/sedentary time and treating these as a continuous variable. We repeated the analyses of the data using ≥1 and ≥ 2 year of follow-up restrictions (‘conventional’ sensitivity analyses) and with no follow-up restriction. Finally, we re-analyzed the physical activity and sedentary exposures in continuous form using restricted cubic splines with 3 knots placed at the 10th, 50th and 90th percentiles. All analyses included 4-year sample weights and accounted for the complex survey design to create nationally representative estimates for the US non-institutionalized civilian population above the age of 40 [12]. The NHANES study was reviewed and approved by the National Center for Health Statistics Research Ethics Review Board and all participants provided signed consent for participation.

## Results

After removing 318 deaths occurring within 5 years after the baseline examination, we included in analysis 3542 participants, of which 489 deceased, with a median (interquartile range) follow-up of 10.8 (9.7 to 11.8) years. Mean (standard deviation) age at baseline was 56.1 (11.4) years and 54% were women. Participant characteristics stratified by quartiles of total physical activity are presented in Table 1.

**Table 1:** Descriptive Characteristics of Participants With ≥5 Years of Follow-up by Total Physical Activity, NHANES, 2003–2006

	Total (*n* = 3542)	Q1 CPM (*n* = 884)	Q2 CPM (*n* = 887)	Q3 CPM (*n* = 885)	Q4 CPM (*n* = 886)
Age (years)	56.1 (11.4)	65.3 (13.6)	56.9 (10.9)	54.4 (9.4)	50.6 (7.7)
Sex (% female)	54	65	60	57	37
BMI kg/m^2^)	28.7 (6.0)	29.9 (7.5)	29.5 (6.7)	28.7 (5.3)	27.2 (4.5)
Overweight (%)	38	38	34	38	41
Obese (%)	34	40	39	34	25
**Education (%)**					
< High School	15	21	14	12	15
High School (including GED)	26	29	26	26	25
> High School	59	50	60	62	61
**Race/ethnicity (%)**					
Mexican-American	5	3	4	6	7
Non-Hispanic White	78	81	78	78	78
Non-Hispanic Black	9	10	10	10	8
Other	7	7	8	7	7
**Smoking (%)**					
Never	49	46	50	52	47
Former	32	36	30	29	33
Current	19	18	20	20	20
**Alcohol consumption (%)**					
Never	10	16	12	11	5
Former	20	28	19	19	15
Current	65	53	64	65	75
Missing	5	4	6	6	4
Married/living with partner (% yes)	73	63	71	75	79
Mobility limitations (% yes)	11	22	13	8	4
No. of medical conditions	0.38 (0.79)	0.78 (1.21)	0.39 (0.79)	0.31 (0.62)	0.17 (0.49)
Total physical activity (CPM)	297 (140)	134 (39)	222 (22)	302 (26)	473 (106)
Light physical activity (min/day)^a^	333 (89)	236 (64)	308 (55)	353 (61)	401 (79)
Moderate-to-vigorous physical activity (min/day)^a^	21.6 (21.3)	3.7 (4.0)	10.9 (6.8)	20.0 (9.4)	45.0 (22.7)
Sedentary time (hrs/day)^a^	8.2 (1.6)	10.1 (1.5)	8.8 (0.9)	7.9 (0.9)	6.7 (1.3)

Mean (SD) unless stated otherwise. *CPM* counts/min, *Q* Quartile, *BMI* Body mass index

^a^Calculated as % wear-time in activity/sedentary domain multiplied with mean wear-time of sample

**Fig. 1:**
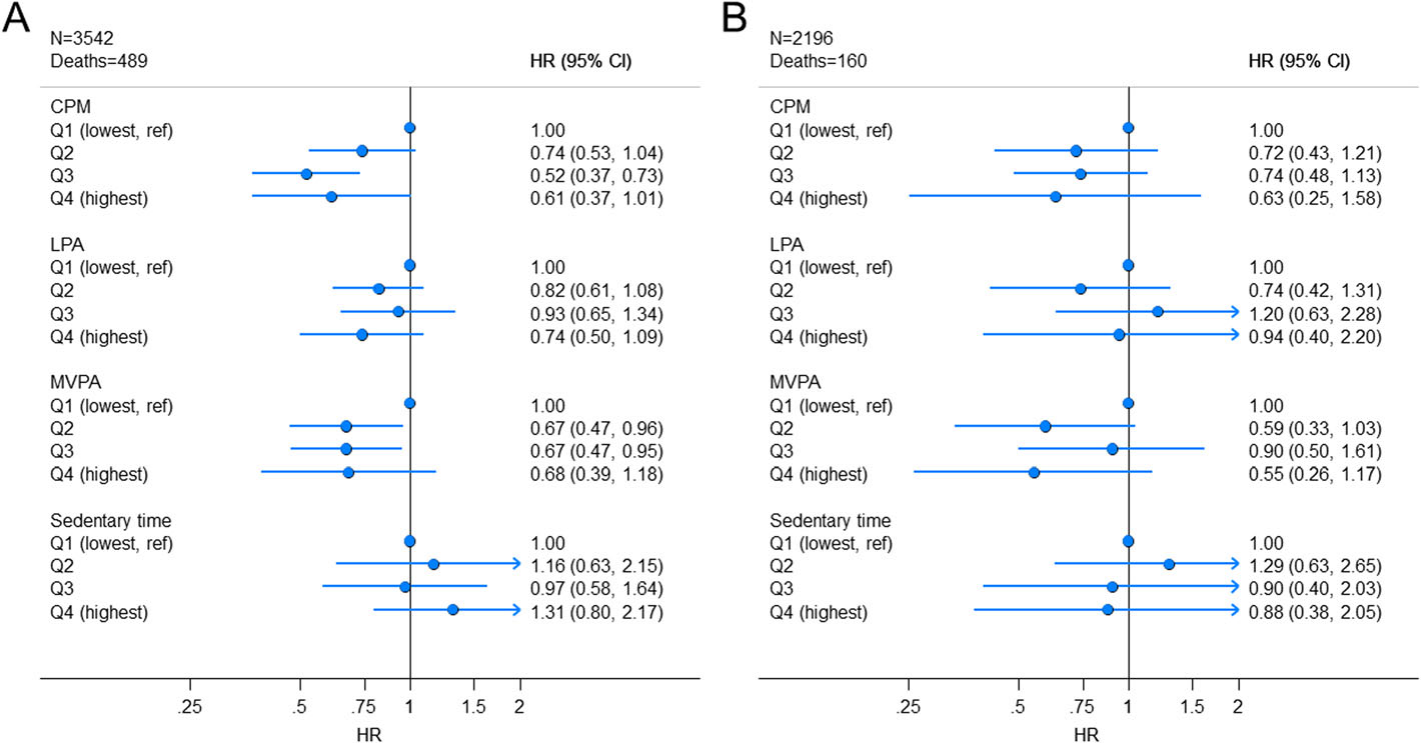
Associations between total and intensity-specific physical activity and sedentary time with all-cause mortality, NHANES, 2003–2006. Panel A: Restricted to individuals with ≥5 years of follow-up. Panel B: As panel A + excluding individuals with mobility limitations and prevalent diabetes, cardiovascular disease, or cancer. Models in both panels adjusted for age (continuous), sex, BMI (continuous), education (<High School, High School (including GED), or > High School), race/ethnicity (Mexican-American, Non-Hispanic White, Non-Hispanic Black, or other), alcohol consumption status (never, former, current or missing), smoking-status (never, former or current), marital status (married/living with partner or widowed/divorced/separated/never married). MVPA and sedentary time models are mutually adjusted. Models in panel A adjusted for mobility limitations (any difficulty walking up ten steps or walking a quarter mile), number of medical conditions (continuous score of diabetes, congestive heart failure, coronary heart disease, angina/angina pectoris, heart attack, stroke, cancer or malignancy). CPM; counts/min, LPA; Light physical activity, MVPA; Moderate-to-vigorous physical activity, Q; quartile. Median activity levels (Panel A) in ascending quartiles are 134 CPM, 223 CPM, 299 CPM, and 444 CPM for total physical activity, 222 mins/day, 299 mins/day, 358 mins/day, and 441 mins/day for LPA, 2.1 mins/day, 8.4 mins/day, 19.2 mins/day, and 41.6 mins/day for MVPA, and 6.1 h/day, 7.7 h/day, 8.9 h/day, and 10.4 h/day for sedentary time

HRs for total physical activity, LPA, and MVPA were consistently below 1 when comparing quartiles 2–4 against the least active reference quartile, indicative of lower mortality risk with higher physical activity after excluding the first 5 years of follow-up. The association was graded for total physical activity (*P* for trend = 0.003) whereas no further gain was evident in quartiles 3 and 4 for MVPA (*P* for trend = 0.28, Fig. 1 and Additional File Table 1). All CIs included 1 for LPA (*P* for trend (0.16). The HRs comparing quartile 4 with quartile 1 were 0.61; (95%CI: 0.38, 1.01) for CPM, 0.74; (95%CI: 0.50, 1.09) for LPA, and 0.68; (95%CI: 0.39, 1.18) for MPVA. The HR for sedentary time comparing quartile 4 with quartile 1 was 1.31; (95%CI: 0.80, 2.17) with no indication of a trend (*P* = 0.25). When further limiting our analysis to 2196 individuals (160 deaths) free of mobility limitations and chronic disease at baseline HRs for total physical activity were materially unchanged but slightly stronger for MVPA, although all CIs were wide and included 1 (Fig. 1 and Additional File Table 2). There was no evidence to suggest LPA or sedentary time were associated with mortality with HRs at 0.94; (95%CI: 0.40, 2.20) for quartile 4 versus quartile 1 for LPA and 0.88 (95%CI: 0.38, 2.05) for sedentary time.

**Fig. 2:**
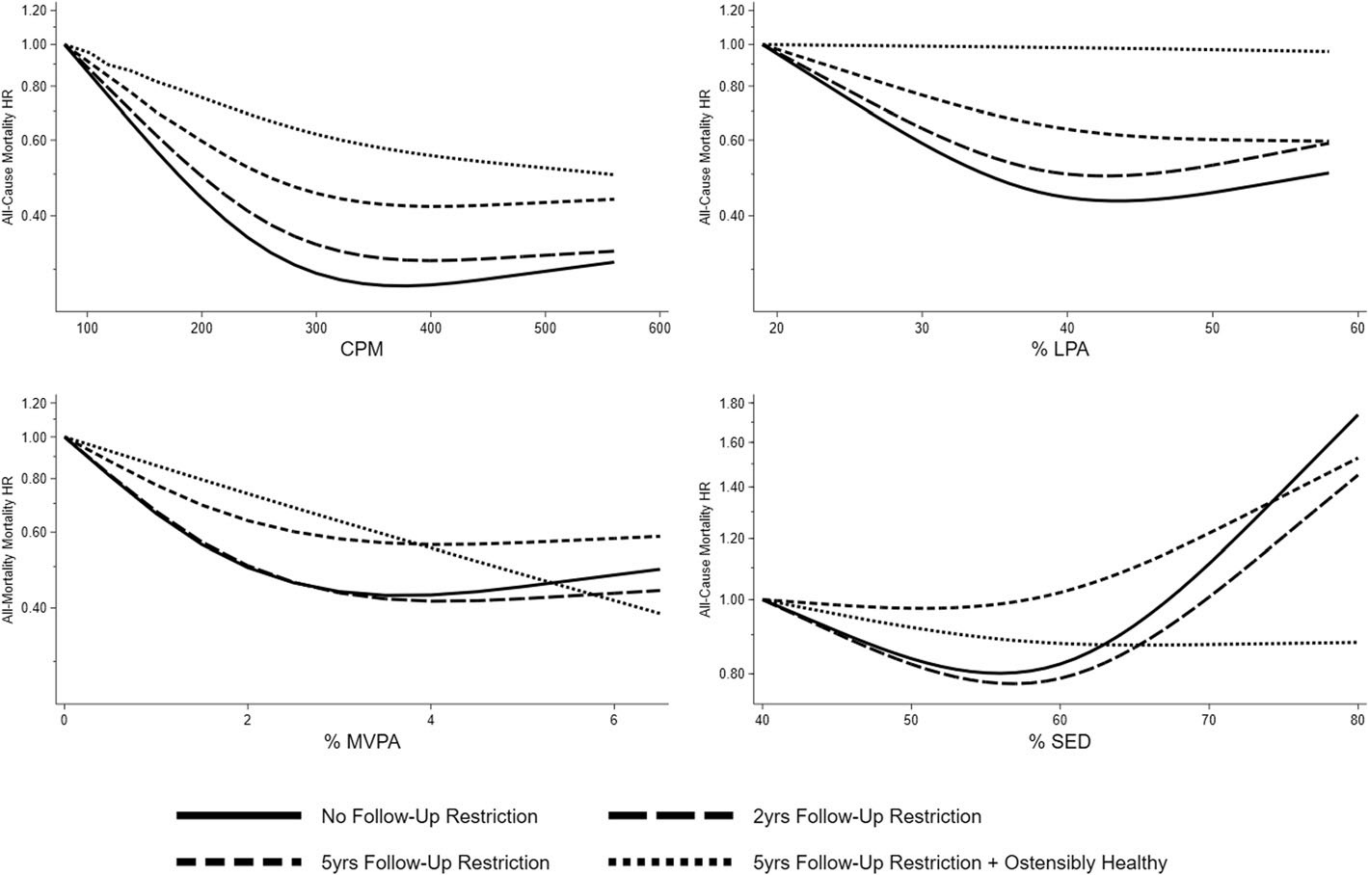
Continuous dose-response associations between physical activity and sedentary time with all-cause mortality, NHANES, 2003–2006. Models adjusted for age (continuous), sex, BMI (continuous), education (<High School, High School (including GED), or > High School), race/ethnicity (Mexican-American, Non-Hispanic White, Non-Hispanic Black, or other), alcohol consumption status (never, former, current or missing), smoking-status (never, former or current), marital status (married/living with partner or widowed/divorced/separated/never married), mobility limitations (any difficulty walking up ten steps or walking a quarter mile), number of medical conditions (continuous score of diabetes, congestive heart failure, coronary heart disease, angina/angina pectoris, heart attack, stroke, cancer or malignancy). MVPA and sedentary time models are mutually adjusted. Analysis in ostensibly healthy individuals excludes participants with baseline mobility limitations or prevalent diabetes, cardiovascular disease, or cancer. Reference levels are placed at the 5th percentile of the exposure distribution in the sample with no follow-up restriction. CPM; counts/min, LPA; Light physical activity, MVPA; Moderate-to-vigorous physical activity

Baseline characteristics stratified by baseline age and years under observation from baseline are shown in Additional File Table 3. Individuals who deceased within 5 years after baseline reported more medical conditions, were less physically active, and more likely to be current smoker across all age-strata. A higher frequency of mobility limitations was observed in the 60–70 years age-stratum. Repeating the analysis with restriction to individuals with follow-up time of ≥1 year (3811 participants, 758 deaths, follow-up of 10.6 (9.5 to 11.7 years) showed, as compared with the ≥5 years restriction, a consistent pattern of greater magnitude of associations for total physical activity, LPA and MVPA with trend *P*-values of < 0.001, 0.01, and 0.008, respectively (Additional File Table 4). The no-association pattern for sedentary time was similar. HRs for quartile 4 versus quartile 1 were 0.49; (95%CI: 0.36, 0.69) for total physical activity, 0.72; (95%CI: 0.52, 1.006) for LPA, and 0.52; (95%CI: 0.35, 0.76) for MVPA. This corresponds to a 25% greater magnitude of association (ratio of HRs of 1.25) for total physical activity and a 31% greater magnitude of association for MVPA comparing top quartiles from the analysis excluding deaths within the first year with the same quartile from analysis excluding deaths within the first 5 years. Analysis restricted to individuals with *≥*2 years of follow-up yielded slightly attenuated HRs, as compared with the *≥*1 year restriction, for total activity and LPA but not for MVPA (Additional File Table 5). Analysis with no restriction on follow-up time is presented in Additional File Table 6. An overview of associations with progressively more conservative restriction criteria is presented in Additional File Figure 2. Figure 2 and Additional File Figures 3–6 presents the dose-response patterns from analysis using physical activity and sedentary time in continuous form. These analyses supported attenuated HRs with increasing restriction of follow-up time with very similar patterns of the associations except when restricting to individuals free from mobility limitations and prevalent diabetes, cardiovascular disease, or cancer.

## Discussion

Our results suggest that total physical activity and MVPA is associated with lower mortality after more than 10 years of follow-up and excluding deaths within the first 5 years to minimize the risk of reverse causation bias from undiagnosed disease. The pattern of associations was in a beneficial direction although some CIs included 1. The association between LPA and mortality was weaker and with all CIs including 1. HRs for total physical activity and MVPA were attenuated as compared with the analyses that excluded deaths within the first year, but still supported a substantially lower risk of mortality with higher levels of physical activity. There was no association between sedentary time and mortality risk after excluding deaths within the first 5 years. There was no pattern of association between LPA and mortality after further removal of participants with mobility limitations or prevalent disease, while associations with total activity and MVPA, albeit attenuated and with wide CIs all crossing unity, remained suggestive of a lower risk of premature mortality.

Our analysis rests on the assumption that individuals with low physical activity levels due to undiagnosed diseases will have died within the first 5 years of follow-up. There is no empirical data to support this assumption and it is possible that an even longer time frame is needed to fully capture the influence of sub-clinical disease on mortality [11, 16]. However, for subclinical disease to manifest in behavioral changes such as reduced physical activity, the disease would probably need to be relatively progressed suggesting a (too) long exclusion window could be overly conservative. We based our decision to exclude mortalities within the first 5 years on the methodology applied in recent reviews of the BMI-mortality association [16, 17]. We consider the biological likelihood of undiagnosed disease impacting BMI and physical activity levels as equally plausible, although others have asserted that the potential benefits of restrictions on alleviating reverse causality bias may be outweighed by unknown validity and reduced generalizability of the analysis [18, 19]. With these uncertainties in mind, we consider our results less susceptible to bias from reverse causation than previous studies using device-based measurements of physical activity and sedentary time, which were able to exclude only the first 1 or 2 years because of relatively few mortalities due to short follow-up time [4–6, 20–24]. As was evident from the attenuation of HRs when restricting to individuals with ≥5 years of follow-up, excluding the first 1 or 2 years of follow-up may not fully remove bias caused by low physical activity levels resulting from undiagnosed disease. The optimal balance between risk of bias from reverse causation and overly conservative follow-up time restriction is unknown and likely specific for the exposure-outcome association in question [11, 25] in addition to characteristics of the target population under study. This was nicely illustrated by a recent study showing that associations between physical inactivity and dementia/Alzheimer’s disease could be completely explained by cases diagnosed within the first 10 years, whereas this was not the case for type 2 diabetes and CVD [25]. The interpretations of the authors were that the association with dementia/Alzheimer’s disease was likely non-causal. Unfortunately, only a crude measure of self-reported physical activity was available. A recent study from the UK Biobank demonstrated that mortality associations with self-reported MVPA differed substantially when the authors expanded their analysis from using 1 to the maximum available 7 years of follow-up [9]. Longer follow-up will certainly reduce the impact of early deaths on measures of association, but a noteworthy observation from the UK Biobank study is that the HRs from the least controlled model (0.86) adjusting for prevalent disease and the most conservative model (0.88) excluding individuals with prevalent disease in addition to the first 2 years of observation, were nearly identical at 7 years of follow-up. However, as the authors only excluded the first 2 years our data suggest the possibility of residual reverse-causality bias in these estimates may remain. We extend the observations in the UK Biobank cohort by; 1) showing that a fairly long follow-up of up to 13 years may not fully minimize the influence of reverse causality bias and needs to be coupled with exclusion of early deaths, and 2) suggesting the influence of reverse-causality bias is intensity-domain specific. Until large cohort with device-based measurement of physical activity have been followed for sufficient time and accrued sufficient cases to minimize the impact of early deaths on effect estimates (which, from this study should be based on informative CIs with exclusions of deaths within at least the first 2 years), we suggest studies report sensitivity analyses with a range of reasonable follow-up time restrictions. In this way, robustness of results can be asserted and it will facilitate meta-analysis of published summary results if a harmonization approach is not possible [26]. As this was an observational study we cannot eliminate the risk of other biases; however, our analyses based on device-measured physical activity coupled with exclusion of deaths occurring within first 5 years provide robustness to the evidence supporting the role of total physical activity and MVPA in reducing the risk of premature mortality. Our results also support the suggestion that the sedentary time-mortality association observed in previous studies is highly influenced by deaths occurring within a short time frame [27]. Longevity may thus be served better by advocating physical activity rather than just limiting total sedentary time. Large cohorts including older individuals with device-based measurements of sedentary time will be in a good position to examine the generalizability of this finding when sufficient follow-up time has been accrued [6, 21, 24].

The introduction of device-based measurements of physical activity in large cohort studies have suggested the reduced risk of mortality is not confined to MVPA, but that LPA may be sufficient and even similar to MVPA in magnitude of the association. The association between LPA and mortality is robust after controlling for MVPA in some [5, 22] but not all [21] studies. We did not find strong evidence to support beneficial associations with LPA in our main analysis and when we further excluded individuals with diagnosed diabetes, cardiovascular diseases or cancers, the association was completely attenuated with a HR for the upper quartile of LPA close to 1 and a completely flat dose-response pattern in the analysis based on LPA modelled as a continuous variable. This may suggest that in apparently healthy middle-aged individuals with expected survival of more than 5 years, physical activity should be of at least moderate intensity (equivalent to walking) [28], whereas e.g. activities of the daily-living (which often falls into the LPA domain) may not be sufficient to reduce mortality risk. Activities of at least moderate intensity may, if performed with sufficient frequency and volume, increase cardiorespiratory and muscular fitness which are strong predictors of mortality and morbidity [29, 30] and may be feasibly evaluated in a clinical setting [31]. These findings should be reproduced in other cohorts but feeds directly into a discussion of the relative importance of total, LPA and MVPA in relation to health and mortality [1]. Importantly, these findings do not preclude benefits of high levels of LPA in populations older than this cohort [5, 6] or in individuals living with mobility limitations, diabetes, CVD or cancer. The 2018 The Physical Activity Guidelines for Americans does not discriminate between healthy individuals and those living with chronic diseases [1] which would suggest these target populations share identical dose-response patterns between physical activity and mortality/morbidity. The substantial change in the observed dose-response patterns, particularly for LPA and sedentary time, when we restricted to individuals with at least 5 years survival who were free of mobility limitations and major chronic diseases at baseline is thus noteworthy. Determining associations between total and intensity-specific physical activities in individuals with and without chronic disease will be an important line of inquiry in future studies.

We highlight the following limitations; 1) the analysis excluding individuals with mobility limitations or prevalent medical conditions had only a modest number of cases and are thus sensitive to chance variation. This is reflected in wide confidence intervals but the patterns of associations, while attenuated, were similar for total activity and MVPA but substantially altered for LPA and sedentary time, 2) although a strength of our study is the long follow-up duration of 13 years and the large number of deaths, with longer follow-up time one may also expect greater change in the exposure over time, leading to misclassification of the exposure, which may bias estimates both towards or away from unity [32]. The behavioral or demographic (e.g. retirement) factors associated with changes in exposure over time may not be identical across physical activity intensity domains, 3) we accounted for prevalent conditions such as diabetes, cardiovascular disease and cancer but did not account for e.g. respiratory or mental diseases which may have impacted HRs, 4) as an observational study the risk of residual or unmeasured confounding and others biases cannot be ruled out. How perfect control of these other biases would modify the impact of reverse-causation bias is unknown, 5) information on recent unintentional weight-loss (a potential marker of subclinical disease) was unavailable, 6) MVPA and sedentary time were mutually adjusted, but we did not account for time spent in all intensities of physical activity. Time-use physical activity epidemiology is a developing field and it will be interesting to follow the impact of different modelling approaches on association-measures as these methods mature [33].

In summary, the association-patterns between total physical activity and MVPA with risk of mortality after more than 10 years of follow-up was inverse and independent of deaths occurring within the first 5 years. These results further strengthen the evidence of substantial mortality benefits with higher levels of physical activity. There was no pattern of lower mortality with higher LPA in healthy, middle-aged individuals surviving for more than 5 years. Limiting sensitivity analyses to excluding deaths occurring within the first 1 or 2 years may not completely minimize the impact of reserve causation bias on estimates of the physical activity-mortality association.

## Supplementary information

**Additional file 1: Figure S1.** Participant flowchart, National Health and Nutrition Examination Survey, 2003–2006. **Table S1.** Associations between total and intensity-specific physical activity and sedentary time with all-cause mortality restricted to individuals with ≥5 years of follow-up, National Health and Nutrition Examination Survey, 2003–2006. **Table S2.** Associations between total and intensity-specific physical activity and sedentary time with all-cause mortality restricted to individuals with ≥5 years of follow-up and no mobility limitations or prevalent diabetes, CVD or cancer, National Health and Nutrition Examination Survey, 2003–2006. **Table S3.** Descriptive Characteristics of Adults ≥40 of age stratified by age and Time from Baseline to Death, National Health and Nutrition Examination Survey, 2003–2006. **Table S4.** Associations between total and intensity-specific physical activity and sedentary time with all-cause mortality restricted to individuals with ≥1 year of follow-up, National Health and Nutrition Examination Survey, 2003–2006. **Table S5.** Associations between total and intensity-specific physical activity and sedentary time with all-cause mortality restricted to individuals with ≥2 year of follow-up, National Health and Nutrition Examination Survey, 2003–2006. **Table S6.** Associations between total and intensity-specific physical activity and sedentary time with all-cause mortality with no follow-up restriction (all participants with available covariates included), National Health and Nutrition Examination Survey, 2003–2006. **Figure S2.** Associations between quartiles of total and intensity-specific physical activity and sedentary time with all-cause mortality by progressively more conservative restriction criteria, National Health and Nutrition Examination Survey, 2003–2006. **Figure S3.** Continuous dose-response association total physical activity and mortality by progressively more conservative restriction criteria, National Health and Nutrition Examination Survey, 2003–2006. **Figure S4.** Continuous dose-response association light-intensity physical activity and mortality by progressively more conservative restriction criteria, National Health and Nutrition Examination Survey, 2003–2006. **Figure S5.** Continuous dose-response association moderate-to-vigorous physical activity and mortality by progressively more conservative restriction criteria, National Health and Nutrition Examination Survey, 2003–2006. **Figure S6.** Continuous dose-response association sedentary time and mortality by progressively more conservative restriction criteria, National Health and Nutrition Examination Survey, 2003–2006.

## Data Availability

The datasets analysed during the current study are available at the Centers for Disease Control and Prevention webpages https://www.cdc.gov/nchs/data-linkage/mortality-public.htm, https://wwwn.cdc.gov/nchs/nhanes/continuousnhanes/default.aspx?BeginYear=2003 and https://wwwn.cdc.gov/nchs/nhanes/continuousnhanes/default.aspx?BeginYear=2005.

## Abbreviations

NHANES: National Health and Nutrition Examination Survey
CPM: counts/minute
LPA: light physical activity
MVPA: moderate-to-vigorous physical activity
BMI: body mass index
HR: hazard ratio
CI: confidence interval
