# Supplementary material for: Accelerometer-measured physical activity and sedentary time in a cohort of US adults followed for up to 13 years: the influence of removing early follow-up on associations with mortality
Source: Int J Behav Nutr Phys Act. 2020 Mar 14;17:39. doi: 10.1186/s12966-020-00945-4 (PMC7071621; doi:10.1186/s12966-020-00945-4)
Supplement: Supplementary file 1 — Additional file 1: Figure S1. Participant flowchart, National Health and Nutrition Examination Survey, 2003–2006. Table S1. Associations between total and intensity-specific physical activity and sedentary time with all-cause mortality restricted to individuals with ≥5 years of follow-up, National Health and Nutrition Examination Survey, 2003–2006. Table S2. Associations between total and intensity-specific physical activity and sedentary time with all-cause mortality restricted to individuals with ≥5 years of follow-up and no mobility limitations or prevalent diabetes, CVD or cancer, National Health and Nutrition Examination Survey, 2003–2006. Table S3. Descriptive Characteristics of Adults ≥40 of age stratified by age and Time from Baseline to Death, National Health and Nutrition Examination Survey, 2003–2006. Table S4. Associations between total and intensity-specific physical activity and sedentary time with all-cause mortality restricted to individuals with ≥1 year of follow-up, National Health and Nutrition Examination Survey, 2003–2006. Table S5. Associations between total and intensity-specific physical activity and sedentary time with all-cause mortality restricted to individuals with ≥2 year of follow-up, National Health and Nutrition Examination Survey, 2003–2006. Table S6. Associations between total and intensity-specific physical activity and sedentary time with all-cause mortality with no follow-up restriction (all participants with available covariates included), National Health and Nutrition Examination Survey, 2003–2006. Figure S2. Associations between quartiles of total and intensity-specific physical activity and sedentary time with all-cause mortality by progressively more conservative restriction criteria, National Health and Nutrition Examination Survey, 2003–2006. Figure S3. Continuous dose-response association total physical activity and mortality by progressively more conservative restriction criteria, National Health and Nutrition Examinati [file 12966_2020_945_MOESM1_ESM.docx]

Additional File 1

**Accelerometer-measured physical activity and sedentary time in a cohort of US adults followed for up to 13 years: the influence of removing early follow-up on associations with mortality**

Additional File Figure 1. Participant flowchart, National Health and Nutrition Examination Survey, 2003-2006.

Additional File Table 1. Associations between total and intensity-specific physical activity and sedentary time with all-cause mortality restricted to individuals with ≥5 years of follow-up, National Health and Nutrition Examination Survey, 2003-2006.

Additional File Table 2. Associations between total and intensity-specific physical activity and sedentary time with all-cause mortality restricted to individuals with ≥5 years of follow-up and no mobility limitations or prevalent diabetes, CVD or cancer, National Health and Nutrition Examination Survey, 2003-2006.

Additional File Table 3. Descriptive Characteristics of Adults ≥40 of age stratified by age and Time from Baseline to Death, National Health and Nutrition Examination Survey, 2003-2006

Additional File Table 4. Associations between total and intensity-specific physical activity and sedentary time with all-cause mortality restricted to individuals with ≥1 year of follow-up, National Health and Nutrition Examination Survey, 2003-2006.

Additional File Table 5. Associations between total and intensity-specific physical activity and sedentary time with all-cause mortality restricted to individuals with ≥2 year of follow-up, National Health and Nutrition Examination Survey, 2003-2006.

Additional File Table 6. Associations between total and intensity-specific physical activity and sedentary time with all-cause mortality with no follow-up restriction (all participants with available covariates included), National Health and Nutrition Examination Survey, 2003-2006.

Additional File Figure 2. Associations between quartiles of total and intensity-specific physical activity and sedentary time with all-cause mortality by progressively more conservative restriction criteria, National Health and Nutrition Examination Survey, 2003-2006

Additional File Figure 3. Continuous dose-response association total physical activity and mortality by progressively more conservative restriction criteria, National Health and Nutrition Examination Survey, 2003-2006.

Additional File Figure 4. Continuous dose-response association light-intensity physical activity and mortality by progressively more conservative restriction criteria, National Health and Nutrition Examination Survey, 2003-2006.

Additional File Figure 5. Continuous dose-response association moderate-to-vigorous physical activity and mortality by progressively more conservative restriction criteria, National Health and Nutrition Examination Survey, 2003-2006.

Additional File Figure 6. Continuous dose-response association sedentary time and mortality by progressively more conservative restriction criteria, National Health and Nutrition Examination Survey, 2003-2006.

Additional File Figure 1. Participant flowchart, National Health and Nutrition Examination Survey, 2003-2006.

Above 40 years old + eligible for mortality follow-up n = 6361

+

At least 4 days of 10+ hours of physical activity data

No PA file = 1214

<4 days with 10 = 827

+

No follow-up = 1

n = 4319

Deaths = 1065

+

Missing co-variables

BMI

Pregnant women = 4

Unable to stand straight = 291

BMI not available = 41

BMI > 100 = 1

Educational attainment = 5

Race/ethnicity = 0

Smoking = 0

Alcohol = 0

Marital status = 2

Mobility status = 56

Diabetes / CVD /cancer history = 62

**n = 3860**

**Deaths = 807**

Excluding individuals with <5 years of follow-up = 318

**n = 3542**

**Deaths = 489**

Excluding individuals with mobility limitations = 470

Excluding individuals with prevalent diabetes, CVD or cancer

= 876

**n = 2196**

**Deaths = 160**

Additional File Table 1. Associations between total and intensity-specific physical activity and sedentary time with all-cause mortality restricted to individuals with ≥5 years of follow-up, National Health and Nutrition Examination Survey, 2003-2006.

|  | Q1 | Q2 | Q3 | Q4 | *P*-trend |
| --- | --- | --- | --- | --- | --- |
| CPM |  |  |  |  |  |
| HR (95%CI) | 1 (ref) | 0.74 (0.53 to 1.04) | 0.52 (0.37 - 0.73) | 0.61 (0.37 - 1.01) | 0.003 |
| N | 884 | 887 | 885 | 886 |  |
| Deaths | 263 | 111 | 66 | 49 |  |
| Person-years | 9083 | 9521 | 9621 | 9602 |  |
| Median CPM | 134 | 223 | 299 | 444 |  |
|  |  |  |  |  |  |
| LPA |  |  |  |  |  |
| HR (95%CI) | 1 (ref) | 0.82 (0.61 - 1.08) | 0.93 (0.65 - 1.34) | 0.74 (0.50 - 1.09) | 0.16 |
| N | 885 | 886 | 885 | 886 |  |
| Deaths | 216 | 114 | 96 | 63 |  |
| Person-years | 9242 | 9548 | 9500 | 9631 |  |
| Median LPA (min/day)* | 226 | 298 | 358 | 438 |  |
|  |  |  |  |  |  |
| MVPA |  |  |  |  |  |
| HR (95%CI) | 1 (ref) | 0.67 (0.47 - 0.96) | 0.67 (0.47 - 0.95) | 0.68 (0.39 - 1.18) | 0.28 |
| N | 885 | 886 | 885 | 886 |  |
| Deaths | 265 | 103 | 69 | 52 |  |
| Person-years | 9075 | 9633 | 9552 | 9572 |  |
| Median MVPA (min/day)* | 2.2 | 8.6 | 19.2 | 40.9 |  |
|  |  |  |  |  |  |
| Sedentary time |  |  |  |  |  |
| HR (95%CI) | 1 (ref) | 1.16 (0.63 - 2.15) | 0.97 (0.58 - 1.64) | 1.31 (0.80 - 2.17) | 0.25 |
| N | 885 | 886 | 885 | 886 |  |
| Deaths | 59 | 89 | 109 | 232 |  |
| Person-years | 9610 | 9581 | 9459 | 9209 |  |
| Median sedentary time (hrs/day)* | 6.3 | 7.8 | 8.9 | 10.2 |  |

N = 3542, deaths = 489. HRs and 95% CIs. *Calculated as % wear-time in intensity domain multiplied with mean wear-time of sample.

Models include; age (continuous), sex, BMI (continuous), education (<High School, High School (including GED), or >High School), race/ethnicity (Mexican-American, Non-Hispanic White, Non-Hispanic Black, or other), alcohol consumption status (never, former, current or missing), smoking-status (never, former or current), marital status (married/living with partner or widowed/divorced/separated/never married), mobility limitations (any difficulty walking up ten steps or walking a quarter mile), number of medical conditions (continuous score of diabetes, congestive heart failure, coronary heart disease, angina/angina pectoris, heart attack, stroke, cancer or malignancy). MVPA and sedentary time models are mutually adjusted. CPM; counts/min, LPA; Light physical activity, MVPA; Moderate-to-vigorous physical activity, Q; quartile

Additional File Table 2. Associations between total and intensity-specific physical activity and sedentary time with all-cause mortality restricted to individuals with ≥5 years of follow-up and no mobility limitations or prevalent diabetes, CVD or cancer, National Health and Nutrition Examination Survey, 2003-2006.

|  | Q1 | Q2 | Q3 | Q4 | *P*-trend |
| --- | --- | --- | --- | --- | --- |
| CPM |  |  |  |  |  |
| HR (95%CI) | 1 (ref) | 0.72 (0.43 - 1.21) | 0.74 (0.48 - 1.13) | 0.63 (0.25 - 1.58) | 0.28 |
| N | 549 | 549 | 549 | 549 |  |
| Deaths | 77 | 35 | 28 | 20 |  |
| Person-years | 5838 | 5963 | 5962 | 5934 |  |
| Median CPM | 172 | 254 | 334 | 476 |  |
|  |  |  |  |  |  |
| LPA |  |  |  |  |  |
| HR (95%CI) | 1 (ref) | 0.74 (0.42 - 1.31) | 1.20 (0.63 - 2.28) | 0.94 (0.40 - 2.20) | 0.90 |
| N | 549 | 549 | 549 | 549 |  |
| Deaths | 57 | 38 | 35 | 30 |  |
| Person-years | 5868 | 5952 | 5950 | 5941 |  |
| Median LPA (min/day)* | 243 | 310 | 371 | 451 |  |
|  |  |  |  |  |  |
| MVPA |  |  |  |  |  |
| HR (95%CI) | 1 (ref) | 0.59 (0.33 - 1.03) | 0.90 (0.50 - 1.61) | 0.55 (0.26 - 1.17) | 0.25 |
| N | 549 | 549 | 549 | 549 |  |
| Deaths | 79 | 31 | 35 | 15 |  |
| Person-years | 5835 | 5945 | 5974 | 5939 |  |
| Median MVPA (min/day)* | 4.0 | 12.2 | 23.7 | 45.2 |  |
|  |  |  |  |  |  |
| Sedentary time |  |  |  |  |  |
| HR (95%CI) | 1 (ref) | 1.29 (0.63 - 2.65) | 0.90 (0.40 - 2.03) | 0.88 (0.38 - 2.05) | 0.50 |
| N | 549 | 549 | 549 | 549 |  |
| Deaths | 25 | 35 | 40 | 60 |  |
| Person-years | 5948 | 5953 | 5940 | 5870 |  |
| Median sedentary time (hrs/day)* | 6.0 | 7.5 | 8.6 | 9.8 |  |

N = 2196, deaths = 160. HRs and 95% CIs. *Calculated as % wear-time in intensity domain multiplied with mean wear-time of sample.

Models include; age (continuous), sex, BMI (continuous), education (<High School, High School (including GED), or >High School), race/ethnicity (Mexican-American, Non-Hispanic White, Non-Hispanic Black, or other), alcohol consumption status (never, former, current or missing), smoking-status (never, former or current), marital status (married/living with partner or widowed/divorced/separated/never married). MVPA and sedentary time models are mutually adjusted. CPM; counts/min, LPA; Light physical activity, MVPA; Moderate-to-vigorous physical activity, Q; quartile

Additional File Table 3. Descriptive Characteristics of Adults ≥40 of age stratified by age and Time from Baseline to Death, National Health and Nutrition Examination Survey, 2003-2006

|  | Deceased  within 1 year | Deceased  within 1 to 2 years | Deceased  within 2 to 5 years | Not deceased  within 5 years | p-value^c^ |
| --- | --- | --- | --- | --- | --- |
| *40-60 years old at baseline* | | | | | |
| n | 4 | 7 | 40 | 1809 |  |
| Age (years) | 54.1 (2.3) | 55.09 (4.8) | 51.5 (5.8) | 49.3 (4.8) | 0.004 |
| BMI kg/m^2^ | 25.9 (1.8) | 26.9 (8.3) | 30.3 (7.1) | 28.9 (5.3) | 0.66 |
| **Smoking (%)** |  |  |  |  | 0.004 |
| Former | 68 | 0 | 30 | 26 |  |
| Current | 13 | 80 | 40 | 23 |  |
| Mobility limitations (% yes) | 13 | 21 | 12 | 7 | 0.13 |
| No. medical condition | 0.68 (1.08) | 2.05 (1.7) | 0.47 (0.70) | 0.22 (0.48) | 0.03 |
| Total physical activity (CPM) | 300 (146) | 201 (164) | 274 (146) | 333 (119) | 0.04 |
| Sedentary time (hrs/day)^a^ | 9.3 (1.0) | 9.6 (1.6) | 8.3 (2.1) | 7.9 (1.3) | 0.09 |
| Primary cause of death was  CVD or cancer (% of deaths) | 52 | 76 | 49 | 49^b^ | Insufficient within-strata variation to perform test |
| *≥60 - 70 years old at baseline* | | | | | |
| n | 14 | 15 | 40 | 904 |  |
| Age (years) | 65.3 (3.6) | 65.1 (3.0) | 65.3 (4.0) | 64.6 (3.3) | 0.11 |
| BMI kg/m^2^ | 31.9 (7.7) | 25.1 (6.2) | 28.9 (7.3) | 29.0 (6.8) | 0.73 |
| **Smoking (%)** |  |  |  |  | 0.03 |
| Former | 53 | 48 | 59 | 41 |  |
| Current | 26 | 20 | 27 | 16 |  |
| Mobility limitations (% yes) | 37 | 47 | 41 | 15 | <0.001 |
| No. medical condition | 1.87 (2.04) | 1.36 (1.50) | 1.15 (2.00) | 0.61 (1.19) | 0.002 |
| Total physical activity (CPM) | 160 (101) | 226 (248) | 166 (97) | 254 (137) | 0.002 |
| Sedentary time (hrs/day)^a^ | 9.9 (1.8) | 9.1 (2.4) | 9.7 (2.1) | 8.6 (1.7) | 0.001 |
| Primary cause of death was  CVD or cancer (% yes) | 79 | 57 | 70 | 46^b^ | Insufficient within-strata variation to perform test |
| *≥70 - 80 years old at baseline* | | | | | |
| n | 12 | 16 | 64 | 574 |  |
| Age (years) | 74.8 (3.4) | 75.4 (2.7) | 75.7 (4.2) | 74.2 (3.3) | 0.002 |
| BMI kg/m^2^ | 25.0 (6.8) | 28.3 (5.7) | 27.3 (7.9) | 27.7 (6.0) | 0.57 |
| **Smoking (%)** |  |  |  |  | <0.001 |
| Former | 38 | 35 | 54 | 48 |  |
| Current | 23 | 32 | 17 | 6 |  |
| Mobility limitations (% yes) | 36 | 40 | 26 | 23 | 0.22 |
| No. medical condition | 1.61 (1.98) | 1.23 (1.40) | 1.29 (1.99) | 0.81 (1.22) | 0.02 |
| Total physical activity (CPM) | 123 (132) | 107 (68) | 146 (105) | 205 (117) | <0.001 |
| Sedentary time (hrs/day)^a^ | 11.0 (2.2) | 10.8 (1.3) | 9.8 (2.3) | 9.1 (1.8) | <0.001 |
| Primary cause of death was  CVD or cancer (% yes) | 44 | 70 | 64 | 38^b^ | Insufficient within-strata variation to perform test |
| *≥80 years old at baseline* | | | | | |
| n | 19 | 17 | 70 | 255 |  |
| Age (years) | 83.9 (2.6) | 83.6 (2.6) | 83.6 (2.6) | 83.1 (2.7) | 0.04 |
| BMI kg/m^2^ | 24.8 (8.1) | 27.6 (4.5) | 25.5 (6.3) | 26.3 (5.6) | 0.34 |
| **Smoking (%)** |  |  |  |  | 0.04 |
| Former | 43 | 39 | 49 | 38 |  |
| Current | 10 | 3 | 6 | 2 |  |
| Mobility limitations (% yes) | 34 | 36 | 27 | 26 | 0.42 |
| No. medical condition | 1.77 (1.92) | 1.12 (0.92) | 1.22 (1.48) | 0.87 (1.55) | 0.002 |
| Total physical activity (CPM) | 97 (75) | 126 (97) | 123 (110) | 153 (116) | 0.001 |
| Sedentary time (hrs/day)^a^ | 10.8 (2.1) | 10.3 (1.9) | 10.4 (2.2) | 9.7 (2.0) | <0.001 |
| Primary cause of death was  CVD or cancer (% yes) | 57 | 56 | 47 | 40^b^ | Insufficient within-strata variation to perform test |

Mean (SD) unless stated otherwise. CPM; counts/min, BMI; Body mass index, CVD; Cardiovascular disease

^a^Calculated as % wear-time in sedentary domain multiplied with mean wear-time of sample.

^b^Evaluated among individuals who deceased >5 years after baseline to end of observation time.

^c^P-value from t-test or chi-squared test contrasting individuals with recorded death within 5 years of baseline versus individuals surviving for at least 5 years after baseline.

Additional File Table 4. Associations between total and intensity-specific physical activity and sedentary time with all-cause mortality restricted to individuals with ≥1 year of follow-up, National Health and Nutrition Examination Survey, 2003-2006.

|  | Q1 | Q2 | Q3 | Q4 | *P*-trend |
| --- | --- | --- | --- | --- | --- |
| CPM |  |  |  |  |  |
| HR (95%CI) | 1 (ref) | 0.60 (0.48 - 0.76) | 0.41 (0.30 - 0.55) | 0.49 (0.36 - 0.69) | <0.001 |
| N | 951 | 951 | 956 | 953 |  |
| Deaths | 410 | 164 | 102 | 82 |  |
| Person-years | 8720 | 9969 | 10271 | 10120 |  |
| Median CPM | 130 | 215 | 294 | 441 |  |
|  |  |  |  |  |  |
| LPA |  |  |  |  |  |
| HR (95%CI) | 1 (ref) | 0.70 (0.54 - 0.92) | 0.68 (0.52 - 0.87) | 0.72 (0.52 - 1.006) | 0.008 |
| N | 952 | 953 | 953 | 953 |  |
| Deaths | 347 | 167 | 141 | 103 |  |
| Person-years | 9193 | 9957 | 10111 | 10156 |  |
| Median LPA (min/day)* | 220 | 293 | 354 | 435 |  |
|  |  |  |  |  |  |
| MVPA |  |  |  |  |  |
| HR (95%CI) | 1 (ref) | 0.60 (0.46 - 0.78) | 0.43 (0.31 - 0.59) | 0.52 (0.35 - 0.76) | 0.008 |
| N | 952 | 953 | 953 | 953 |  |
| Deaths | 414 | 166 | 95 | 83 |  |
| Person-years | 8602 | 10095 | 10221 | 10177 |  |
| Median MVPA (min/day)* | 1.8 | 7.5 | 18.0 | 40.2 |  |
|  |  |  |  |  |  |
| Sedentary time |  |  |  |  |  |
| HR (95%CI) | 1 (ref) | 0.89 (0.57 - 1.39) | 0.85 (0.57 - 1.28) | 1.23 (0.86 - 1.76) | 0.06 |
| N | 952 | 953 | 953 | 953 |  |
| Deaths | 97 | 131 | 164 | 366 |  |
| Person-years | 10146 | 10155 | 9991 | 9059 |  |
| Median sedentary time (hrs/day)* | 6.4 | 7.9 | 8.9 | 10.3 |  |

N = 3811, deaths = 758. HRs and 95% CIs. *Calculated as % wear-time in intensity domain multiplied with mean wear-time of sample.

Models include; age (continuous), sex, BMI (continuous), education (<High School, High School (including GED), or >High School), race/ethnicity (Mexican-American, Non-Hispanic White, Non-Hispanic Black, or other), alcohol consumption status (never, former, current or missing), smoking-status (never, former or current), marital status (married/living with partner or widowed/divorced/separated/never married), mobility limitations (any difficulty walking up ten steps or walking a quarter mile), number of medical conditions (continuous score of diabetes, congestive heart failure, coronary heart disease, angina/angina pectoris, heart attack, stroke, cancer or malignancy). MVPA and sedentary time models are mutually adjusted. CPM; counts/min, LPA; Light physical activity, MVPA; Moderate-to-vigorous physical activity, Q; quartile

Additional File Table 5. Associations between total and intensity-specific physical activity and sedentary time with all-cause mortality restricted to individuals with ≥2 year of follow-up, National Health and Nutrition Examination Survey, 2003-2006.

|  | Q1 | Q2 | Q3 | Q4 | *P*-trend |
| --- | --- | --- | --- | --- | --- |
| CPM |  |  |  |  |  |
| HR (95%CI) | 1 (ref) | 0.67 (0.51 - 0.87) | 0.46 (0.34 - 0.62) | 0.54 (0.38 - 0.76) | <0.001 |
| N | 939 | 938 | 937 | 942 |  |
| Deaths | 377 | 153 | 97 | 76 |  |
| Person-years | 8955 | 9868 | 10092 | 10105 |  |
| Median CPM | 133 | 217 | 295 | 441 |  |
|  |  |  |  |  |  |
| LPA |  |  |  |  |  |
| HR (95%CI) | 1 (ref) | 0.78 (0.60 - 1.02) | 0.74 (0.55 - 0.997) | 0.80 (0.57 - 1.11) | 0.06 |
| N | 939 | 939 | 939 | 939 |  |
| Deaths | 315 | 159 | 131 | 98 |  |
| Person-years | 9307 | 9892 | 9992 | 10028 |  |
| Median LPA (min/day)* | 222 | 294 | 355 | 436 |  |
|  |  |  |  |  |  |
| MVPA |  |  |  |  |  |
| HR (95%CI) | 1 (ref) | 0.64 (0.47 - 0.86) | 0.47 (0.34 - 0.64) | 0.54 (0.36 - 0.81) | 0.01 |
| N | 939 | 939 | 939 | 939 |  |
| Deaths | 379 | 156 | 92 | 76 |  |
| Person-years | 8835 | 10003 | 10080 | 10053 |  |
| Median MVPA (min/day)* | 1.9 | 7.8 | 18.1 | 40.3 |  |
|  |  |  |  |  |  |
| Sedentary time |  |  |  |  |  |
| HR (95%CI) | 1 (ref) | 0.92 (0.58 - 1.46) | 0.78 (0.53 - 1.14) | 1.18 (0.80 - 1.74) | 0.19 |
| N | 939 | 939 | 939 | 939 |  |
| Deaths | 90 | 128 | 146 | 339 |  |
| Person-years | 10033 | 10026 | 9924 | 9183 |  |
| Median sedentary time (hrs/day)* | 6.4 | 7.9 | 8.9 | 10.3 |  |

N = 3756, deaths = 703. HRs and 95% CIs. *Calculated as % wear-time in intensity domain multiplied with mean wear-time of sample.

Models include; age (continuous), sex, BMI (continuous), education (<High School, High School (including GED), or >High School), race/ethnicity (Mexican-American, Non-Hispanic White, Non-Hispanic Black, or other), alcohol consumption status (never, former, current or missing), smoking-status (never, former or current), marital status (married/living with partner or widowed/divorced/separated/never married), mobility limitations (any difficulty walking up ten steps or walking a quarter mile), number of medical conditions (continuous score of diabetes, congestive heart failure, coronary heart disease, angina/angina pectoris, heart attack, stroke, cancer or malignancy). MVPA and sedentary time models are mutually adjusted. CPM; counts/min, LPA; Light physical activity, MVPA; Moderate-to-vigorous physical activity, Q; quartile

Additional File Table 6. Associations between total and intensity-specific physical activity and sedentary time with all-cause mortality with no follow-up restriction (all participants with available covariates included), National Health and Nutrition Examination Survey, 2003-2006.

|  | Q1 | Q2 | Q3 | Q4 | *P*-trend |
| --- | --- | --- | --- | --- | --- |
| CPM |  |  |  |  |  |
| HR (95%CI) | 1 (ref) | 0.58 (0.46 - 0.72) | 0.41 (0.30 - 0.57) | 0.48 (0.34 - 0.69) | <0.001 |
| N | 965 | 965 | 965 | 965 |  |
| Deaths | 441 | 174 | 106 | 86 |  |
| Person-years | 8568 | 10082 | 10350 | 10294 |  |
| Median CPM | 126 | 213 | 293 | 439 |  |
|  |  |  |  |  |  |
| LPA |  |  |  |  |  |
| HR (95%CI) | 1 (ref) | 0.69 (0.53 - 0.91) | 0.66 (0.52 - 0.84) | 0.66 (0.48 - 0.93) | 0.002 |
| N | 965 | 965 | 964 | 966 |  |
| Deaths | 375 | 179 | 146 | 107 |  |
| Person-years | 9094 | 10019 | 10205 | 10285 |  |
| Median LPA (min/day)* | 218 | 292 | 354 | 434 |  |
|  |  |  |  |  |  |
| MVPA |  |  |  |  |  |
| HR (95%CI) | 1 (ref) | 0.61 (0.48 - 0.78) | 0.46 (0.34 - 0.62) | 0.55 (0.37 - 0.83) | 0.03 |
| N | 965 | 965 | 963 | 967 |  |
| Deaths | 442 | 176 | 100 | 89 |  |
| Person-years | 8508 | 10141 | 10313 | 10291 |  |
| Median MVPA (min/day)* | 1.8 | 7.3 | 17.7 | 40.1 |  |
|  |  |  |  |  |  |
| Sedentary time |  |  |  |  |  |
| HR (95%CI) | 1 (ref) | 0.90 (0.57 - 1.44) | 0.91 (0.59 - 1.39) | 1.31 (0.91 - 1.89) | 0.02 |
| N | 965 | 965 | 965 | 965 |  |
| Deaths | 100 | 137 | 175 | 395 |  |
| Person-years | 10282 | 10238 | 10075 | 8938 |  |
| Median sedentary time (hrs/day)* | 6.4 | 7.9 | 9.0 | 10.3 |  |

N = 3860, deaths = 807. HRs and 95% CIs. *Calculated as % wear-time in intensity domain multiplied with mean wear-time of sample.

Models include; age (continuous), sex, BMI (continuous), education (<High School, High School (including GED), or >High School), race/ethnicity (Mexican-American, Non-Hispanic White, Non-Hispanic Black, or other), alcohol consumption status (never, former, current or missing), smoking-status (never, former or current), marital status (married/living with partner or widowed/divorced/separated/never married), mobility limitations (any difficulty walking up ten steps or walking a quarter mile), number of medical conditions (continuous score of diabetes, congestive heart failure, coronary heart disease, angina/angina pectoris, heart attack, stroke, cancer or malignancy). MVPA and sedentary time models are mutually adjusted. CPM; counts/min, LPA; Light physical activity, MVPA; Moderate-to-vigorous physical activity, Q; quartile

Additional File Figure 2. Associations between quartiles of total and intensity-specific physical activity and sedentary time with all-cause mortality by progressively more conservative restriction criteria, National Health and Nutrition Examination Survey, 2003-2006


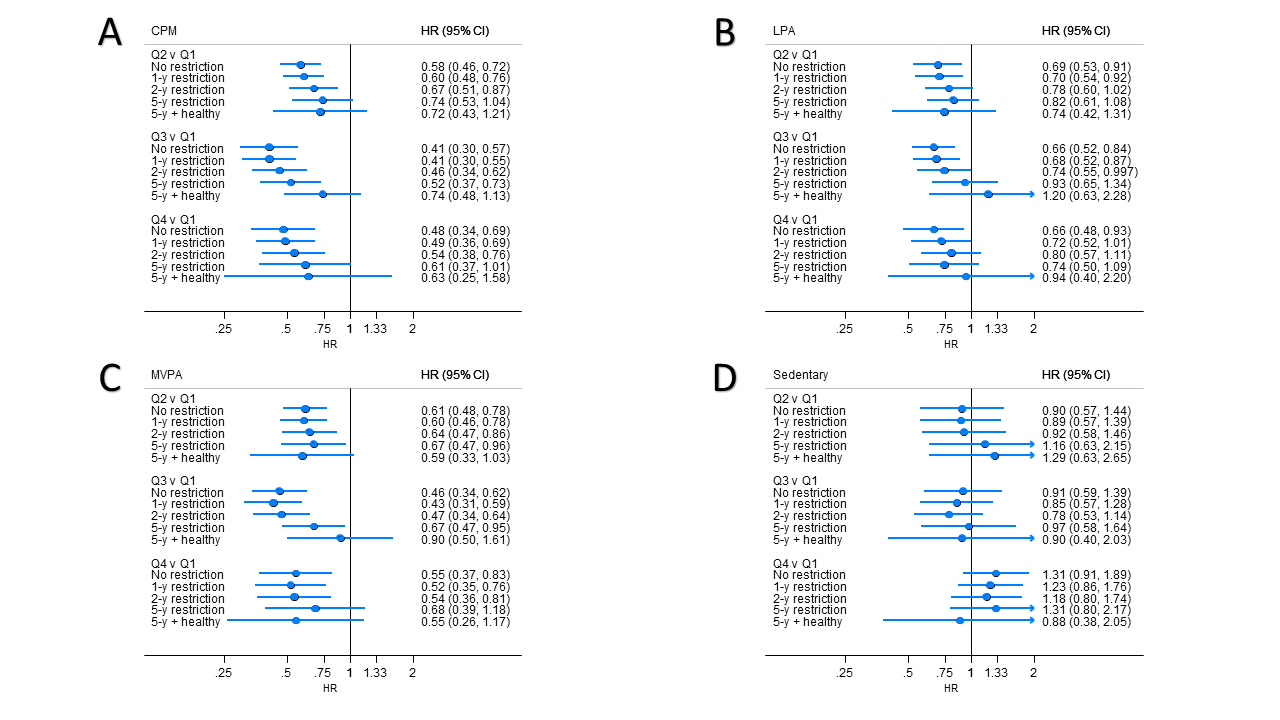


Panels A-D are total physical activity (CPM), LPA, MVPA, and sedentary time, respectively. All HRs in reference to the intensity- and restriction criterion specific least active quartile (least sedentary quartile for sedentary time). Models adjusted for age (continuous), sex, BMI (continuous), education (<High School, High School (including GED), or >High School), race/ethnicity (Mexican-American, Non-Hispanic White, Non-Hispanic Black, or other), alcohol consumption status (never, former, current or missing), smoking-status (never, former or current), marital status (married/living with partner or widowed/divorced/separated/never married), mobility limitations (any difficulty walking up ten steps or walking a quarter mile), number of medical conditions (continuous score of diabetes, congestive heart failure, coronary heart disease, angina/angina pectoris, heart attack, stroke, cancer or malignancy). MVPA and sedentary time models are mutually adjusted. Individuals with mobility limitations and prevalent diabetes, cardiovascular disease, or cancer are excluded from models restricted to healthy individuals with ≥5 years of observation time. CPM; counts/min, LPA; Light physical activity, MVPA; Moderate-to-vigorous physical activity, Q; quartile

Additional File Figure 3. Continuous dose-response association total physical activity and mortality by progressively more conservative restriction criteria, National Health and Nutrition Examination Survey, 2003-2006.

Models adjusted for age (continuous), sex, BMI (continuous), education (<High School, High School (including GED), or >High School), race/ethnicity (Mexican-American, Non-Hispanic White, Non-Hispanic Black, or other), alcohol consumption status (never, former, current or missing), smoking-status (never, former or current), marital status (married/living with partner or widowed/divorced/separated/never married), mobility limitations (any difficulty walking up ten steps or walking a quarter mile), number of medical conditions (continuous score of diabetes, congestive heart failure, coronary heart disease, angina/angina pectoris, heart attack, stroke, cancer or malignancy). Analysis in ostensibly healthy individuals excludes participants with baseline mobility limitations or prevalent diabetes, cardiovascular disease, or cancer. Reference levels are placed at the 5^th^ percentile of the exposure distribution in the sample with no follow-up restriction. CPM; counts/min

Additional File Figure 4. Continuous dose-response association light-intensity physical activity and mortality by progressively more conservative restriction criteria, National Health and Nutrition Examination Survey, 2003-2006.

Models adjusted for age (continuous), sex, BMI (continuous), education (<High School, High School (including GED), or >High School), race/ethnicity (Mexican-American, Non-Hispanic White, Non-Hispanic Black, or other), alcohol consumption status (never, former, current or missing), smoking-status (never, former or current), marital status (married/living with partner or widowed/divorced/separated/never married), mobility limitations (any difficulty walking up ten steps or walking a quarter mile), number of medical conditions (continuous score of diabetes, congestive heart failure, coronary heart disease, angina/angina pectoris, heart attack, stroke, cancer or malignancy). Analysis in ostensibly healthy individuals excludes participants with baseline mobility limitations or prevalent diabetes, cardiovascular disease, or cancer. Reference levels are placed at the 5^th^ percentile of the exposure distribution in the sample with no follow-up restriction. LPA; Light-intensity physical activity

Additional File Figure 5. Continuous dose-response association moderate-to-vigorous physical activity and mortality by progressively more conservative restriction criteria, National Health and Nutrition Examination Survey, 2003-2006.

Models adjusted for age (continuous), sex, BMI (continuous), sedentary time (continuous), education (<High School, High School (including GED), or >High School), race/ethnicity (Mexican-American, Non-Hispanic White, Non-Hispanic Black, or other), alcohol consumption status (never, former, current or missing), smoking-status (never, former or current), marital status (married/living with partner or widowed/divorced/separated/never married), mobility limitations (any difficulty walking up ten steps or walking a quarter mile), number of medical conditions (continuous score of diabetes, congestive heart failure, coronary heart disease, angina/angina pectoris, heart attack, stroke, cancer or malignancy). Analysis in ostensibly healthy individuals excludes participants with baseline mobility limitations or prevalent diabetes, cardiovascular disease, or cancer. Reference levels are placed at the 5^th^ percentile of the exposure distribution in the sample with no follow-up restriction. MVPA; Moderate-to-vigorous physical activity

Additional File Figure 6. Continuous dose-response association sedentary time and mortality by progressively more conservative restriction criteria, National Health and Nutrition Examination Survey, 2003-2006.

Models adjusted for age (continuous), sex, BMI (continuous), moderate-to-vigorous physical activity (continuous) education (<High School, High School (including GED), or >High School), race/ethnicity (Mexican-American, Non-Hispanic White, Non-Hispanic Black, or other), alcohol consumption status (never, former, current or missing), smoking-status (never, former or current), marital status (married/living with partner or widowed/divorced/separated/never married), mobility limitations (any difficulty walking up ten steps or walking a quarter mile), number of medical conditions (continuous score of diabetes, congestive heart failure, coronary heart disease, angina/angina pectoris, heart attack, stroke, cancer or malignancy). Analysis in ostensibly healthy individuals excludes participants with baseline mobility limitations or prevalent diabetes, cardiovascular disease, or cancer. Reference levels are placed at the 5^th^ percentile of the exposure distribution in the sample with no follow-up restriction.
